# Heterogeneity in the developmental potential of motor neuron progenitors revealed by clonal analysis of single cells *in vitro*

**DOI:** 10.1186/1749-8104-4-2

**Published:** 2009-01-05

**Authors:** Dritan Agalliu, Ira Schieren

**Affiliations:** 1 Department of Biochemistry and Molecular Biophysics, Howard Hughes Medical Institute, Columbia University Medical Center, New York, NY 10032, USA; 2 Department of Neurobiology, Stanford University School of Medicine, Stanford, CA 94305, USA

## Abstract

**Background:**

The differentiation of neural progenitors into distinct classes within the central nervous system occurs over an extended period during which cells become progressively restricted in their fates. In the developing spinal cord, Sonic Hedgehog (Shh) controls neural fates in a concentration-dependent manner by establishing discrete ventral progenitor domains characterized by specific combinations of transcription factors. It is unclear whether motor neuron progenitors can maintain their identities when expanded *in vitro *and whether their developmental potentials are restricted when exposed to defined extracellular signals.

**Results:**

We have generated mice expressing the enhanced green fluorescent protein under the control of the *Nkx6.1 *promoter, enabling fluorescence-activated cell sorting (FACS), purification and culture of individual spinal progenitors at clonal density, and analysis of their progeny. We demonstrate that cells isolated after progenitor domains are established are heterogeneous with respect to maintaining their identity after *in vitro *expansion. Most Nkx6.1^+ ^progenitors lose their ventral identity following several divisions in culture, whereas a small subset is able to maintain its identity. Thus, subtype-restricted progenitors from the Nkx6.1^+ ^region are present in the ventral spinal cord, although at a lower frequency than expected. Clones that maintain a motor neuron identity assume a transcriptional profile characteristic of thoracic motor neurons, despite some having been isolated from non-thoracic regions initially. Exposure of progenitors to Bone Morphogenetic Protein-4 induces some dorsal cell type characteristics in their progeny, revealing that lineage-restricted progenitor subtypes are not fully committed to their fates.

**Conclusion:**

These findings support a model whereby continuous Shh signaling is required to maintain the identity of ventral progenitors isolated from the spinal cord, including motor neuron progenitors, after *in vitro *expansion. They also demonstrate that pre-patterned neural progenitors isolated from the central nervous system can change their regional identity *in vitro *to acquire a broader developmental potential.

## Background

The cellular diversity of the vertebrate central nervous system (CNS) relies upon the generation of distinct neuronal subclasses at defined positions and times from a relatively small pool of proliferating progenitors. As neural progenitors proliferate, they are exposed to secreted inductive signals that initiate cell fate decisions by regulating expression of transcription factors. These transcription factors, in turn, impose developmental restrictions on multipotent progenitor cells before ultimately effecting their final differentiation [1-3]. Understanding how extracellular and cell-intrinsic mechanisms are coordinated during CNS development is important not only for understanding embryonic patterning but also for gaining insight into the developmental potential of neuronal stem cells and progenitors isolated from different regions of the CNS [4].

Neural progenitors from different CNS regions exhibit varying degrees of restriction during their development. Heterochronic transplantation studies have revealed that in the cortex and retina, where neurons are born in a temporal order, progenitors acquire critical aspects of their phenotype during their final cell division [5-7]. Young cortical progenitors, which normally generate deep layer neurons in their normal environment, can respond to signals from an older host environment and generate superficial layer neurons [8,9]. Similarly, young retinal progenitors are multipotent and can adopt cell fates characteristic of the host environment, whereas older progenitors are somewhat limited in their developmental potential [10,11]. However, time-lapse lineage analysis *in vitro *has revealed that even young cortical or retinal progenitors have the intrinsic potential to recapitulate the correct sequence of laminar identities when grown as single progenitors in culture [12-14]. Although clonal analysis has also revealed the importance of cell-intrinsic mechanisms for regulating progenitor cell fate decisions, the degree to which the intrinsic program can be modified by extrinsic cues has not been rigorously tested because the identities of the inductive signals as well as molecular markers for distinct progenitors are poorly defined.

In the vertebrate spinal cord, the signals involved in the conversion of progenitor cells to distinct neuronal subtypes have been defined [1,15,16], making it an excellent system to address if and when motor neuron (MN)-restricted progenitors arise during development and whether they can be isolated in culture. Moreover, the purification of MN-restricted cells has important implications for therapeutic efforts to treat neurodegenerative diseases that affect MNs. During spinal cord development, Sonic Hedgehog (Shh), secreted from the notochord and floor plate, induces the expression of several class II homeodomain (HD) proteins primarily belonging to the *Nkx *gene family (e.g. Nkx6.1/6.2/2.2) in a concentration-dependent manner within ventral neural progenitors [17-19]. The class II HD factors repress other class I HD proteins (for example, Pax7, Pax6, Dbx2 and Irx3) in ventral neural progenitors to establish, refine, and stabilize distinct progenitor domains [20]. Nkx6.1 protein is expressed throughout the ventral third of the neural tube, spanning three ventral progenitor domains: p3, pMN and p2. From these domains arise the V3 interneurons, MNs and V2 interneurons, respectively. Genetic studies have revealed an essential role for Nkx6.1 in MN and V2 interneuron fates, through repression of Dbx2 and establishment of a ventral region of the neural tube [17,21]. Establishment of the Nkx6.1^+ ^region is followed by the expression of 'subtype determinants' that define specific progenitor domains and coordinate neuronal specification and differentiation [22]. These subtype determinants include two closely related Nkx repressor proteins (Nkx2.2/2.9) that are expressed by p3 progenitors and specify V3 neurons [19], as well as the basic helix-loop-helix (bHLH) protein Olig2, which is restricted to pMN progenitors and coordinates MN fate [23,24]. Therefore, labeling progenitors using the Nkx6.1 regulatory elements would allow the isolation and purification of MN and ventral interneuron progenitors.

Despite their uniform generation from the same progenitor domain within the spinal cord, MNs differ along the rostrocaudal axis by expression of Hox proteins that govern their acquisition of distinct motor columnar identities and ultimately their innervation of a variety of targets, such as limbs, intercostals muscles or sympathetic ganglia [25-27]. The expression of HoxC6 by brachial MNs, Hoxc9 by thoracic MNs and Hoxd10 by lumbar MNs depends on graded Fibroblast Growth Factor (FGF) signaling from Hensen's node [25-27]. These transcription factors ensure that distinct MNs acquire lateral motor column identities at limb levels and a preganglionic, fate at the thoracic level, respectively [25-29]. Therefore, graded activities of Shh along the dorsoventral axis and FGFs along the rostrocaudal axis initiate expression of distinct transcriptional programs that are required for generation of generic and columnar identities of MNs.

Despite our understanding of the molecular mechanisms that contribute to the establishment of ventral progenitor and neuronal fates, several issues pertaining to progenitor fate assignment remain unresolved. First, it is unclear whether all cells that express similar levels of transcription factors within a given progenitor domain are able to maintain their identities in a cell-autonomous manner when deprived of endogenous environmental cues. Second, it has not been established whether all pre-patterned MN progenitors exhibit restrictions in their developmental potential, and what the range of neuronal fates are that they can acquire when exposed to defined signals. Neural tissue explants are heterogeneous, which precludes an analysis of progenitor specification at the single-cell level. Third, if MN-restricted progenitors are present in the spinal cord, are they able to proliferate and generate MNs for a prolonged period in culture? Finally, what is the rostrocaudal identity of MNs born from a restricted progenitor in culture? Motor neurons that have been derived *in vitro *from embryonic stem cells share several features with those developing *in vivo*. However, embryonic stem cell-derived MNs have a cervical identity and do not form functional synapses with limb muscles [30]. Isolating lineage-restricted progenitors that can generate MNs in culture may provide an alternative way to produce MNs that maintain their regional identity.

To address these questions, we have generated mice expressing enhanced green fluorescent protein (eGFP) under the control of the *Nkx6.1 *promoter to genetically label ventral progenitors. We prospectively isolated ventral progenitor cells from this strain by fluorescence-activated cell sorting (FACS), cultured individual Nkx6.1^+ ^progenitors at clonal density, and analyzed the molecular identities of their progeny. We demonstrate that ventral Nkx6.1^+ ^cells isolated after progenitor domains are established are heterogeneous in their ability to maintain their identity in culture. The majority of cultured progenitors lose their ventral identity after successive cell divisions without acquiring dorsal fates. A small subset of progenitors, including pMN progenitors, is able to maintain its identity, suggesting that subtype-restricted progenitors from the Nkx6.1^+ ^region are present in the ventral spinal cord, although at a lower frequency than expected from the apparently uniform expression of transcription factors within these progenitors. The pMN-restricted clones have a Hox profile characteristic of thoracic MNs, despite their origin from either forelimb or thoracic levels. The fraction of subtype-restricted progenitors increases over time, suggesting that neural progenitors become progressively more independent of patterning signals. However, exposure of subtype-restricted cells to signals that specify dorsal fates leads to acquisition of some dorsal characteristics, revealing that lineage-restricted progenitor subtypes are not committed to their fate. These findings support a model whereby continuous Shh signaling is required to stably maintain progenitor domain identity after isolation and *in vitro *expansion.

## Results

### Generation of *Nkx6.1::IRES::eGFP *mice and prospective FACS isolation of Nkx6.1^+ ^ventral progenitors

**Figure 1:**
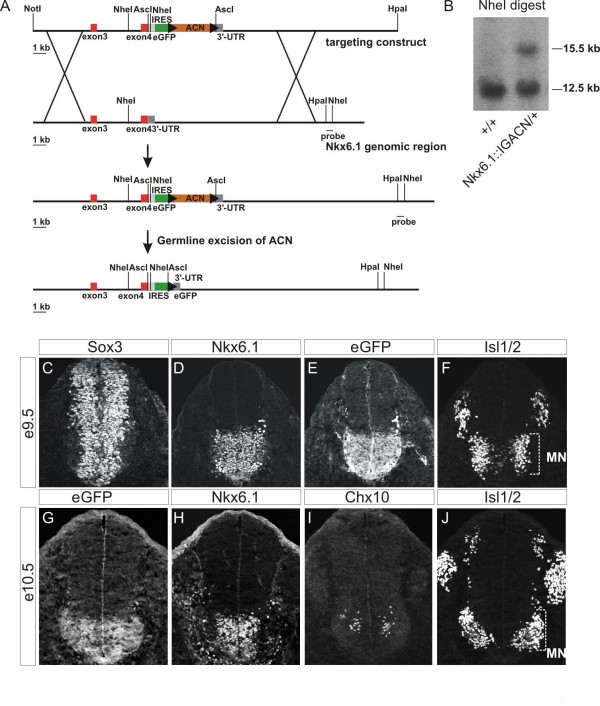
**Generation of *Nkx6.1::IRES::eGFP *mice and embryonic expression of eGFP in the spinal cord**. **(A) **Targeting strategy for the generation of *Nkx6.1::IRES::eGFP *knock-in mice. **(B) **Southern blotting to determine wild type (12.5 kb) and targeted (15.5 kb) alleles at the Nkx6.1 locus. **(C-F) **Expression of Sox3, Nkx6.1, eGFP and Isl1/2 proteins in the e9.5 neural tube of *Nkx6.1::IRES::eGFP*^+/- ^mice. Nkx6.1 and eGFP proteins are expressed in Sox3^+ ^progenitors (C-E). eGFP is also expressed by MNs (F; ventral Isl1/2^+ ^cells). **(G-J) **eGFP is expressed in Nkx6.1^+ ^neural progenitors (G, H) and in mature ventral neuronal subtypes such as V2a interneurons (I; Chx10^+^ cells), and MNs (J; ventral Isl1/2^+^ cells) at e10.5.

We genetically labeled ventral progenitors expressing Nkx6.1, in order to prospectively isolate cells by FACS and determine the degree of their fate restriction and commitment. We inserted an internal ribosome entry site (IRES) followed by the coding region of eGFP after the stop codon of the *Nkx6.1 *gene to allow regulated eGFP expression that faithfully recapitulates the endogenous Nkx6.1 pattern (Figure 1A,B). These mice express eGFP in Nkx6.1^+ ^neural progenitors at embryonic day 9.5 (e9.5). These progenitors are located in the ventral region of the neural tube encompassing three domains (p2, pMN and p3) and express the progenitor marker Sox3 [31] (Figure 1C–E). In addition, eGFP was detected in newly born Isl1/2^+ ^MNs (Figure 1F). The eGFP expression was maintained in Nkx6.1^+ ^progenitors at later stages of development from e10.5 until e13.5 (Figure 1G,H; data not shown) and in all neuronal classes that normally arise from Nkx6.1^+ ^progenitors; namely the V2 and V3 interneurons and MNs (Figure 1G–J; data not shown).

**Figure 2:**
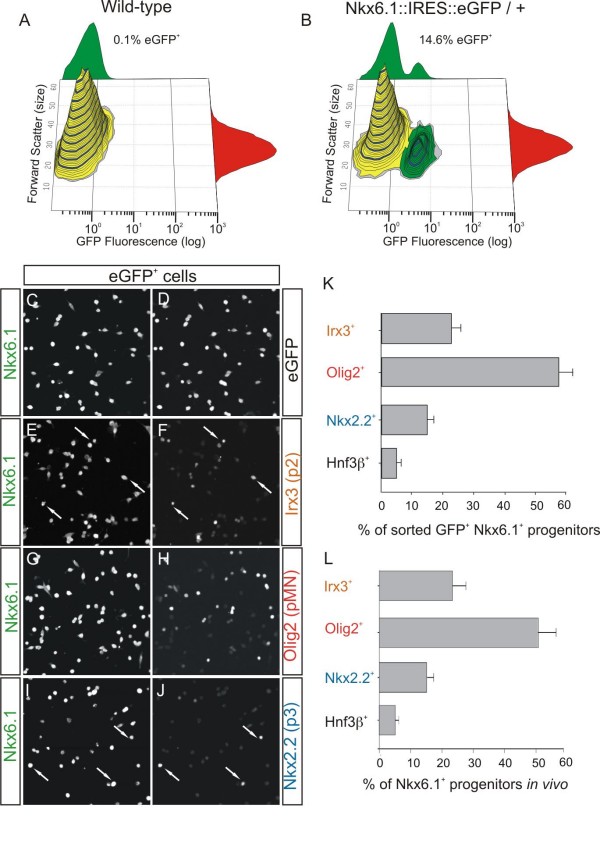
**Sorted eGFP^+ ^ventral progenitors from *Nkx6.1::IRES::eGFP *mice maintain regional identity markers immediately after plating**. **(A, B) **Contour plots of dissociated cells from e9.5 trunks of wild-type (A) or *Nkx6.1::IRES::eGFP*^+/- ^mice (B). The logarithmic scale of eGFP fluorescence is on the x-axis and cell size on the y-axis (forward scatter). **(C-J) **Immunohistochemical analysis of sorted eGFP^+ ^cells after attachment (approximately 2 hours after plating) with antisera for eGFP (D) and transcription factors expressed in different ventral progenitor domains, such as Nkx6.1 (C, E, G, I), Olig2 (H), Irx3 (F) and Nkx2.2 (J). Arrows point to progenitors from the p2 or p3 domains. **(K) **Proportion of sorted eGFP^+^Nkx6.1^+ ^progenitors that express the pMN, p2, p3 or floor plate markers 2 hours after plating. (n = 20 wells from 2 experiments). **(L) **Proportions of three progenitor populations and floor plate within the Nkx6.1^+ ^domain of e9.5 neural tube from 6 sections of brachial and thoracic segments (n = 3 animals). Note that the motor neuron progenitor population (pMN) is the most abundant. Bars represent mean ± s.e.m in all plots.

We determined the purity and molecular profile of sorted eGFP^+ ^cells after FACS purification by analyzing the expression of various transcription factors that are normally found within the Nkx6.1^+ ^region. We isolated eGFP^+ ^cells from neural tubes and somites (trunks) at e9.5, when dorsoventral patterning of neural progenitors is established [20] and MN generation has begun [32]. We found that 14.6% of cells from e9.5 forelimb and thoracic regions expressed eGFP (Figure 2A,B). We analyzed the identities of these cells 2 hours after plating with the following markers: Irx3 (p2), Olig2 (pMN), Nkx2.2 (p3) and Hnf3β (floor plate). We found that 96% of sorted cells were eGFP^+ ^and approximately 65% of the eGFP^+ ^cells expressed the progenitor markers Sox3 and Nkx6.1 (Figure 2C,D; data not shown). The remaining 35% of the GFP^+ ^cells were Hb9^+ ^and Isl1/2^+ ^MNs (data not shown), isolated due to the perdurance of eGFP protein in these cells. The majority of sorted Nkx6.1^+ ^progenitors were pMN progenitors (approximately 58%) that expressed the bHLH protein Olig2 (Figure 2G,H,K). The rest of the progenitors fell into three classes: Irx3^+ ^p2 progenitors (approximately 22%), Nkx2.2^+ ^p3 progenitors (approximately 15%) and HNF3β^+ ^floor plate cells (approximately 5%; Figure 2E–K; data not shown). Therefore, the majority of sorted eGFP^+ ^cells were precursors (65%), of which pMN progenitors comprised the largest population (37%), followed by p2 (14%), and p3 (10%) progenitors (Figure 2K). The fractions of sorted progenitor subtypes were similar to the relative sizes of the p2, pMN and p3 domains within the Nkx6.1^+ ^region *in vivo *(Figure 2L). Therefore, the progenitor populations derived from the Nkx6.1^+ ^domain were faithfully represented in our culture system.

### Non-proliferating Nkx6.1^+ ^neural progenitors differentiate into appropriate neuronal subtypes

**Figure 3:**
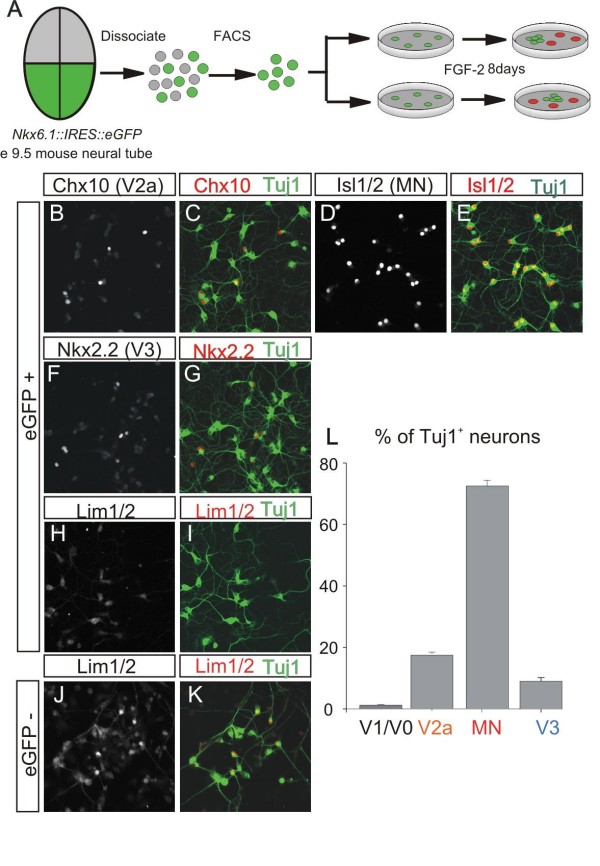
**Sorted Nkx6.1^+ ^progenitors that immediately differentiate *in vitro *generate the correct ventral neuronal subtypes**. **(A) **Schematic diagram of the fluorescence-activated cell sorting (FACS), isolation and culture of Nkx6.1^+ ^spinal progenitors. **(B-G) **Immunohistochemical analysis of neurons that differentiate *in vitro *without proliferation using Chx10 (V2a interneurons; B-C); Isl1/2 (MNs; D-E); and Nkx2.2 (V3 interneurons; F-G) antibodies. Tuj1 stains the neuron-specific βIII tubulin. **(H, I) **No Lim1/2^+ ^neurons were present in cultures from sorted eGFP^+ ^cells, although these neurons were born from sorted eGFP-negative progenitors **(J, K)**. **(L) **Frequencies of three different neuronal subtypes generated *in vitro *from sorted Nkx6.1^+ ^precursors. Bars represent mean ± s.e.m (n = 16 wells from 3 experiments).

In order to test whether pre-patterned ventral spinal progenitors have the ability to maintain their transcriptional identities when expanded *in vitro*, we cultured eGFP^+ ^cells at clonal density in Terasaki microwell plates (6–8 cells/well) for 8 days in a serum-free medium containing FGF2 (Figure 3A). Under these conditions, approximately 84% of Nkx6.1^+ ^progenitors (90% of plated eGFP^+ ^cells) differentiated into Tuj1^+ ^neurons within 24 to 48 hours. This large proportion of progenitors undergoing differentiation *in vitro *could either be due to the dissociation *per se*, which may induce premature differentiation, or may in fact represent normal developmental timing. Because the majority of MN and ventral interneuron differentiation during mouse spinal cord development occurs between e9.5 and e11.5 [32], it is likely that the *in vitro *differentiation of Nkx6.1^+ ^progenitors represents a normal developmental process.

We examined the molecular identity of neurons born from non-proliferating precursors using markers for the three classes of neurons arising from the Nkx6.1^+ ^region: MNs (Hb9, Isl1/2); V2a interneurons (Chx10); and V3 interneurons (Nkx2.2 – a progenitor marker that transiently persists in mature neurons). To facilitate this analysis, we plated eGFP^+ ^sorted cells at higher density (50 cells/well), because their proliferation and differentiation was not affected by the plating density. We found that 72% of differentiated Tuj1^+ ^neurons were Hb9^+ ^and Isl1/2^+ ^MNs, whereas Chx10^+ ^V2a interneurons and Nkx2.2^+ ^V3 interneurons represented 18% and 10% of the total neuronal population, respectively (Figure 3B–G,L). We did not detect any Lim1/2^+ ^neurons, which are normally born from the Nkx6.1-negative dorsal progenitors [33,34], in our cultures (Figure 3H,I,L). However, Lim1/2^+ ^neurons were present in cultures of eGFP-negative neural progenitors (Figure 3J,K). These findings demonstrate that non-proliferating neural progenitors have the ability to generate appropriate neuronal subtypes in the absence of extracellular signals such as Shh and retinoic acid. Therefore, patterning signals that induce class I and class II HD proteins in progenitors are not required for their differentiation.

### Motor neuron and ventral interneuron progenitors are heterogeneous in their ability to maintain dorsoventral transcriptional identities during *in vitro *expansion

**Figure 4:**
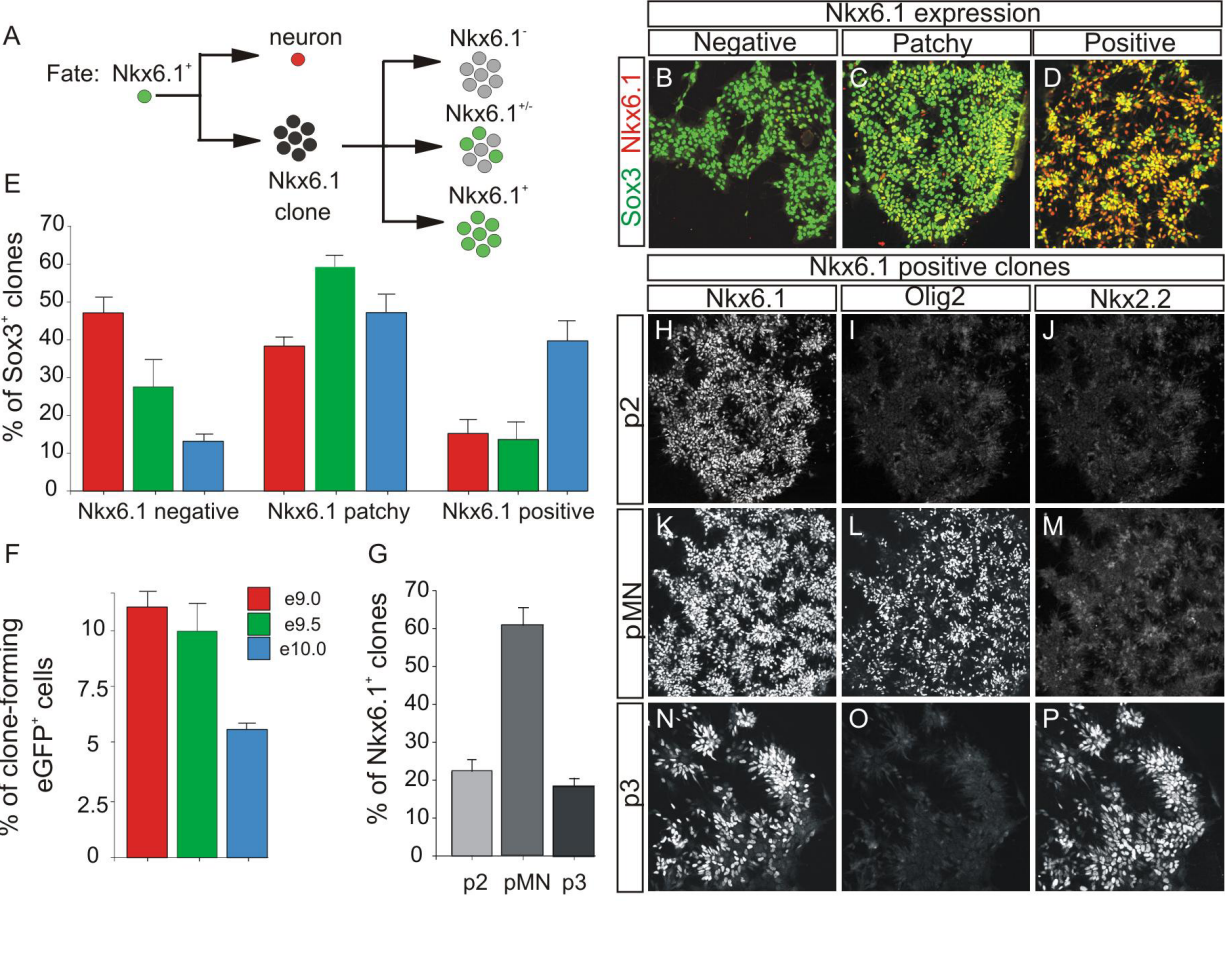
**Ventral subtype-restricted progenitors are present at low frequencies in the spinal cord**. **(A) **Diagram of sorted Nkx6.1^+ ^progenitor fates after culture for several days. **(B-D) **Immunohistochemical analysis for Sox3 and Nkx6.1 in clones derived from single Nkx6.1^+ ^proliferating progenitors. The progenitor state of the cell is revealed by Sox3 (green). Negative clones have no expression of Nkx6.1 (B), patchy clones have some cells that express Nkx6.1 (C; yellow cells) and positive clones have more than 95% of cells that express Nkx6.1 (D). **(E) **Fractions of three different types of clones observed in cultures from sorted e9.0 (red), e9.5 (green) or e10.0 (blue) Nkx6.1^+ ^progenitors. (n = 420 clones from 6 experiments at e9.5; n = 234 clones at e9.0; and n = 124 clones at e10.0 from 3 experiments for the latter two time points). **(F) **Fraction of eGFP^+ ^cells that forms clones in culture at three developmental stages. **(G) **Frequency plot for the three classes of subtype-restricted clones isolated from Nkx6.1^+ ^progenitors (n = 52 Nkx6.1^+ ^clones). Bars represent mean ± s.e.m in all plots. **(H-P) **Expression of Nkx6.1, Olig2 and Nkx2.2 in subtype-restricted progenitors derived from a presumed p2 (H-J), pMN (K-M) or p3 (N-P) progenitor. No clones displayed a mixture of Olig2^+ ^and Nkx2.2^+ ^progenitors.

We next asked if sorted progenitors are able to maintain their transcriptional identities when they proliferate *in vitro *to form clones from a single cell. We cultured single progenitors at clonal density and followed them over the course of 8 days in culture (DIV). We then analyzed the transcriptional identity of clones that were born from a single progenitor. Only a fraction (16%) of Nkx6.1^+ ^progenitors (10% of eGFP^+ ^cells) divided for more than 8 days in culture to generate clones containing precursor cells and neurons (Figure 4A,F). These clones ranged in size from fewer than 20 cells to more than 800 cells, and displayed variable expression of Nkx6.1 protein among Sox3^+ ^progenitors (Figure 4B–D). Based on the frequency of Nkx6.1 expression, we classified these clones into three categories: 'Nkx6.1-negative clones', where proliferating Sox3^+ ^progenitors had lost expression of Nkx6.1 and other ventral progenitor markers, which constituted approximately 28% of the clones (Figure 4B,E; data not shown); 'Nkx6.1-patchy clones', in which a subset of Sox3^+ ^progenitors (ranging between 5% and 95%) expressed Nkx6.1 protein, which were present at the highest frequency in these cultures (approximately 59%; Figure 4C,E); and 'Nkx6.1-positive clones' that maintained expression of Nkx6.1 in more than 95% of Sox3^+ ^progenitors, which were found at the lowest frequency (approximately 13%) (Figure 4D,E). Therefore, a small subset (approximately 13%) of Nkx6.1^+ ^proliferating progenitors is able to maintain its ventral identity *in vitro*, in the absence of signals that induce ventral cell fates.

Next, we analyzed the molecular identity of progenitors and neurons within Nkx6.1-patchy and -positive clones to determine if the expression of progenitor and neuronal subtype transcription factors was maintained. We found that most Nkx6.1-patchy clones were generated from a pMN progenitors because the Nkx6.1^+ ^daughter cells within these clones expressed Olig2 (Figure S1A,B in Additional file 1). We examined the expression of several HD transcription factors characteristic of more dorsal spinal progenitors (e.g. Nkx6.2, Dbx1/2, Pax7) to determine if Nkx6.1-negative daughter cells within Nkx6.1-patchy clones have acquired more dorsal fates. However, none of these factors was expressed in Nkx6.1-negative progenitors (Figure S1C-H in Additional file 1). Therefore, pMN progenitors that lose their ventral identities do not acquire dorsal identities in culture.

We then examined the identity of neurons present in Nkx6.1 and Olig2-patchy clones. HB9^+ ^MNs in these clones represented only a subset (ranging between 5% and 60%) of the total number of Tuj1^+ ^neurons (Figure S1I-K in Additional file 1). The Hb9-negative neurons in these clones did not express markers for dorsal neuronal subtypes such as Lmx1b, Lim1/2, Isl1/2 alone or Lhx2/9 [16] (Figure S1L-N in Additional file 1; data not shown). To extend our observations, we dissected ventral spinal cords from *Hb9::eGFP *mice [30], dissociated them into single cells and cultured the resulting progenitors using our previously established conditions so that we could identify in live clones MNs arising from pMN progenitors, by virtue of their neuronal eGFP expression (Figure 7A). Approximately 60% of proliferating pMN progenitors generated eGFP^+ ^MNs only during the first 2–3 days *in vitro *(DIV), whereas neurons that were born subsequently were eGFP^- ^and did not express markers for dorsal neuronal subtypes such as Lmx1b, Lim1/2, Isl1/2 alone or Lhx2/9 (Figure S1O-Q in Additional file 1; data not shown). Therefore, in Nkx6.1 and Olig2-patchy MN clones, the initial progenitor gradually loses its identity as it undergoes cell division and differentiation.

**Figure 5:**
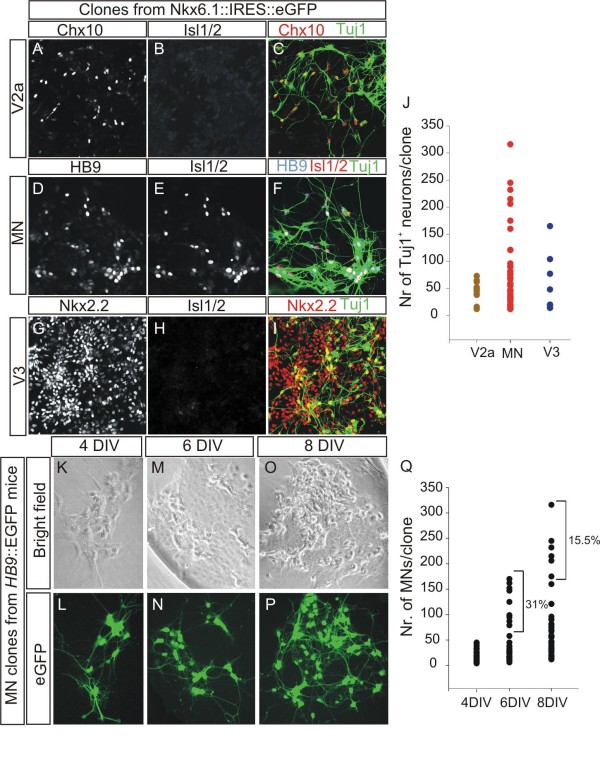
**Clones derived from subtype-restricted ventral progenitors generate appropriate neuronal subtypes *in vitro*. (A-I) **Immunohistochemical analysis of neuronal subtypes present in clones of ventral restricted progenitors with Chx10 (A), Hb9 (D), Isl1/2 (B, E, H) and Nkx2.2 (G) and Tuj1; (C, F, I) are merged panels with Tuj1 to label neurons. **(J) **Vertical dot plot of the number of neurons present in each neuronal subtype restricted clone: Chx10^+ ^V2a interneurons (n = 11 clones), Hb9+ and Isl1/2+ MNs (n = 45 clones) and Nkx2.2^+ ^V3 interneurons (n = 8 clones). **(K-P) **Representative images (bright phase and eGFP) of four- (K, L), six- (M, N) and eight- (O, P) day-old MN clones generated from a pMN-restricted progenitor derived from *Hb9::eGFP *transgenic mice. **(Q) **Vertical dot plot of neuronal number present in pMN-restricted clones from *Hb9::eGFP *mice after four (n = 25 clones), six (n = 32 clones) and eight (n = 45 clones) DIV. The percentage represents the fraction of clones that had more neurons than those counted two days before.

Next, we analyzed the molecular identity of progenitors and neuronal subtypes within the Nkx6.1-positive clones. These clones were further segregated into three distinct types that expressed either the p3 domain marker Nkx2.2 (Figure 4N–P), the pMN domain marker Olig2 (Figure 4K–M) or neither of these markers (Figure 4H–J) in all progenitors. The third type of clone did not express markers characteristic of more dorsal progenitors such as Nkx6.2, Dbx1/2, or Pax7 (data not shown). We did not find any clones that expressed both Nkx6.1 and Irx3, which is characteristic of the p2 domain (data not shown) [20], nor mixed clones where progenitors expressed markers from two ventral domains. We then asked if the molecular identities of neurons present in these three types of Nkx6.1-positive clones correlated with those of neurons born from these three domains in the spinal cord. We found that the p3 domain clones contained exclusively Nkx2.2-expressing Tuj1^+ ^neurons, but not Isl1/2^+ ^or Hb9^+ ^neurons (Figure 5G–I). In contrast, clones derived from a pMN progenitor had only Hb9^+ ^and Isl1/2^+ ^MNs (Figure 5D–F). The third type of clone, expressing only Nkx6.1 but not Irx3 contained Chx10^+ ^neurons, but not Isl1/2^+ ^neurons (Figure 5A–C) and was, therefore, most similar to a V2a interneuron identity [35]. Based on these findings, we presume that the third class is derived from p2 progenitors that have lost expression of Irx3 during proliferation, which does not affect the generation of V2a neurons *in vivo *[36]. In addition, we did not find neurons with mixed subtype identities in any Nkx6.1-positive clones that we examined during this analysis. Therefore, there is a complete match between progenitor and neuronal subtype identity within the Nkx6.1-positive clones, indicating that they were derived from lineage-restricted progenitors that are present in the three domains that express Nkx6.1. These three different clone types were present at distinct frequencies, with MN clones being the most frequent (approximately 60%), followed by V2 clones (approximately 22%) and V3 clones (approximately 18%) (Figure 4G). These proportions correspond to the initial abundance of each sorted precursor type (Figure 2K). Therefore, based on the molecular characterization of progenitors and neuronal subtypes, as well as the proportion of clones belonging to each lineage, we conclude that lineage-restricted progenitor subtypes are present in each progenitor domain of the Nkx6.1^+ ^region in the spinal cord, although at a lower frequency than expected assuming that progenitors derived from the same domain are homogeneous at the time of isolation.

### The frequency of progenitors that maintain their dorsoventral identity after *in vitro *expansion changes over time

We hypothesized that the heterogeneous behavior of Nkx6.1^+ ^progenitors after *in vitro *expansion could result from a differential dependence on inductive signals that establish ventral patterning in the spinal cord. We decided to test whether the subtype frequency of clones that are derived from sorted Nkx6.1^+ ^ventral progenitors changes when progenitors are isolated at different times. Therefore, we FACS isolated Nkx6.1^+ ^progenitors from either e9.0 trunks, when dorsoventral patterning has just begun but no MNs are formed (Figure S2B,C in Additional file 2; data not shown), or from e10.0 trunks, when the majority of MNs are generated (Figure S2E,F in Additional file 2; data not shown). Approximately 5.9% of cells from e9.0 trunks expressed eGFP (Figure S2A in Additional file 2), whereas the proportion of eGFP^+ ^cells was approximately 18% from e10.0 trunks (Figure S2D in Additional file 2). The fraction of sorted eGFP^+ ^cells that proliferated in culture was similar between e9.0 (approximately 12%) and e9.5 (approximately 10%), whereas a smaller number of these cells (approximately 5%) generated clones by e10.0 (Figure 4F), consistent with the *in vivo *timing of ventral neuronal differentiation. Moreover, the frequency of the three clone types that arose *in vitro *also changed over time. The fraction of Nkx6.1-negative clones decreased gradually over time. These clones were present at highest frequency at e9.0 (approximately 48%), whereas by e10.0 they represented the smallest fraction of clones (approximately 12%) (Figure 4E). In contrast, the distribution of Nkx6.1-positive clones over time showed the opposite trend. At e9.0 and e9.5 the frequency of these clones was approximately 14% and approximately 13%, respectively, whereas these clones were very abundant by e10.0 (approximately 40%) (Figure 4E). Finally, the Nkx6.1-patchy clones were less frequent at e9.0 (approximately 38%) when compared to e9.5 (approximately 59%) or e10.0 (approximately 48%) (Figure 4E). These findings indicate that Nkx6.1-negative and Nkx6.1-patchy clones are likely derived from progenitors that are exposed to extracellular signals for a shorter time than progenitors that generate Nkx6.1-positive clones. We have tried to culture sorted Nkx6.1^+ ^progenitors in the presence of various Shh agonist (ShhAg1.3) concentrations [37] to determine if all clones would express Nkx6.1 under these conditions. However, Shh induced differentiation of these cells and no clones were generated (DA and IS, unpublished data). These findings suggest that progenitors giving rise to Nkx6.1-positive clones are likely to be independent of Shh with respect to maintaining their ventral identity, whereas those that generate Nkx6.1-patchy and Nkx6.1-negative clones show varying degrees of Shh dependence.

### Lineage-restricted pMN progenitors have limited neurogenic capacity

We next asked if progenitors from the p2, pMN or p3 domains are able to propagate and generate neurons over extended periods in culture. We counted the number of neurons present at 8 DIV in clones derived from the three different subtypes of lineage-restricted progenitors, in two independent experiments. The number of neurons in each clone was variable but most clones contained between 10 and 80 neurons (Figure 5J). A small fraction (approximately 15.6%) of MN clones that contained a high number of proliferating precursors had a large number of Hb9^+ ^and Isl1/2^+ ^neurons (more than 200 neurons). We then determined the timing of neuronal birth from pMN-restricted progenitors by following the formation of MNs at 4, 6, 8 and 12 days, using clones derived from pMN progenitors that had been isolated from *Hb9*::*eGFP *transgenic mice. We compared the number of MNs in these clones at three different DIV. All clones that we followed were relatively small by 4 DIV and contained between 6 and 48 neurons (Figure 5K,L,Q). After 6 DIV, only a subset of clones (31%) continued to generate MNs (Figure 5M,N,Q) and by 8 days, the fraction of MN-producing clones was further reduced (15.5%) (Figure 5O–Q). After 12 days in culture, no new MNs were born from the pMN-derived clones, but these clones generated a few O4^+ ^oligodendrocytes (Figure S3 in Additional file 3). These observations are similar to those obtained from single cortical neural progenitor cultures *in vitro*, which generate neurons for a limited number of divisions before switching to generate glia [12]. In addition, we were unable to generate MNs from secondary progenitors obtained by passage and expansion of primary MN clones that were first grown for 8 DIV. Taken together, these data indicate that pMN-restricted progenitors lose the ability to generate neurons over time when expanded *in vitro*.

### Brachial- or thoracic-derived pMN progenitors differ in maintenance of rostrocaudal identity after *in vitro *expansion

**Figure 6:**
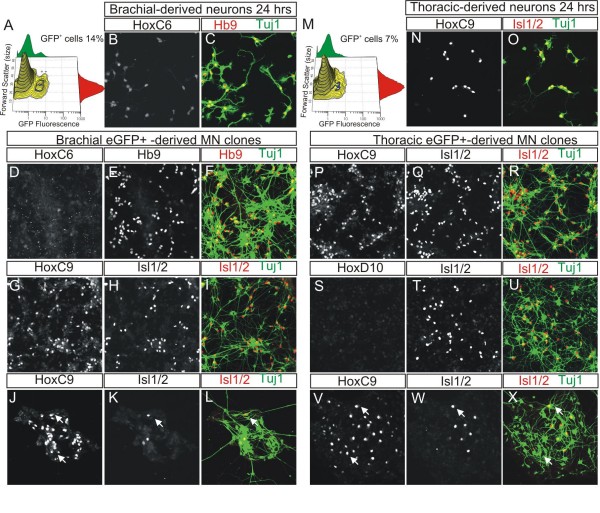
**pMN-restricted and pMN-patchy clones acquire thoracic identity after *in vitro *expansion, regardless of their origin of isolation. (A, M) **Contour plots of dissociated cells from e9.5 brachial (A) or thoracic (M) trunks from *Nkx6.1::IRES::eGFP*^+/- ^mice. **(B, C) **Immunohistochemistry with HoxC6 (B) and Hb9 (red) and Tuj1 (green) (C) of MNs born from non-proliferating brachial Nkx6.1^+ ^progenitors. These motor neurons express low levels of HoxC6. **(D-I) **Immunohistochemistry for HoxC6 (D) or HoxC9 (G) of brachial pMN-restricted clones that contain either Hb9^+^Tuj1^+ ^(E, F) or Isl1/2^+^Tuj1^+ ^(H, I) neurons. Brachial-derived pMN-restricted clones lose expression of HoxC6 and now express HoxC9. **(J-L) **Brachial-derived pMN-patchy clones express HoxC9 (J) even in neurons that have lost their ventral identity (white arrows, K, L). **(N, O) **Immunohistochemistry with HoxC9 (N) and Isl1/2 (red) and Tuj1 (green) (O) of MNs born from non-proliferating thoracic Nkx6.1^+ ^progenitors. These MNs express HoxC9. **(P-U) **Immunohistochemistry for HoxC9 (P) or HoxD10 (S) of thoracic pMN-restricted clones containing Isl1/2^+ ^Tuj1^+ ^neurons. These neurons do not express HoxD10, a marker of lumbar MNs. **(V-X) **Immunohistochemistry for HoxC9 (V) of thoracic pMN-patchy clones that express Isl1/2 (W, X; red) in a subset of Tuj1^+ ^neurons (X; green). Thoracic-derived pMN-patchy clones (white arrows) express HoxC9 even in neurons that have lost their ventral identity.

The lack of expression within Nkx6.1-patchy clones of HD transcription factors that are characteristic of more dorsal progenitor and neuronal subtypes (in Additional file 1) raises the question of whether these clones have acquired rostrocaudal identities that are incompatible with the generation of spinal neurons. To test this possibility, we analyzed the rostrocaudal identities of MNs in lineage-restricted and patchy clones arising from either brachial or thoracic segments of the neural tube at e9.5. Motor neurons that were born from non-proliferating Nkx6.1^+ ^progenitors isolated from either brachial or thoracic regions retained expression of HoxC6 or HoxC9 (Figure 6A–C,M–O), two transcription factors that are normally expressed in MNs born from the brachial or thoracic region, respectively [25,27]. However, the expression of HoxC6 was absent in pMN-patchy or restricted clones that were obtained after *in vitro *expansion of sorted brachial Nkx6.1^+ ^progenitors (Figure 6D–F; data not shown). Moreover, these clones expressed HoxC9 in neurons regardless of their ability to retain or loose their ventral identities (Figure 6G–L). In contrast, pMN-patchy or restricted clones born from *in vitro *expansion of sorted thoracic Nkx6.1^+ ^progenitors maintained expression of the thoracic marker HoxC9 (Figure 6P–R,V–X) and did not express HoxD10, a marker of lumbar MN identity (Figure 6S–U) [28,29]. Therefore, all pMN-derived clones isolated from either brachial or thoracic segments of the neural tube acquire a thoracic identity after *in vitro *expansion, regardless of their ability to maintain their ventral identity. These findings indicate that the Nkx6.1 patchy clones retain their spinal identity and are therefore able to generate neurons from more dorsal sites of origin, but fail to do so in the absence of a dorsal fate-inducing signal.

### pMN-restricted progenitors are not committed to a motor neuron fate when exposed to a dorsally derived inductive signal

**Figure 7:**
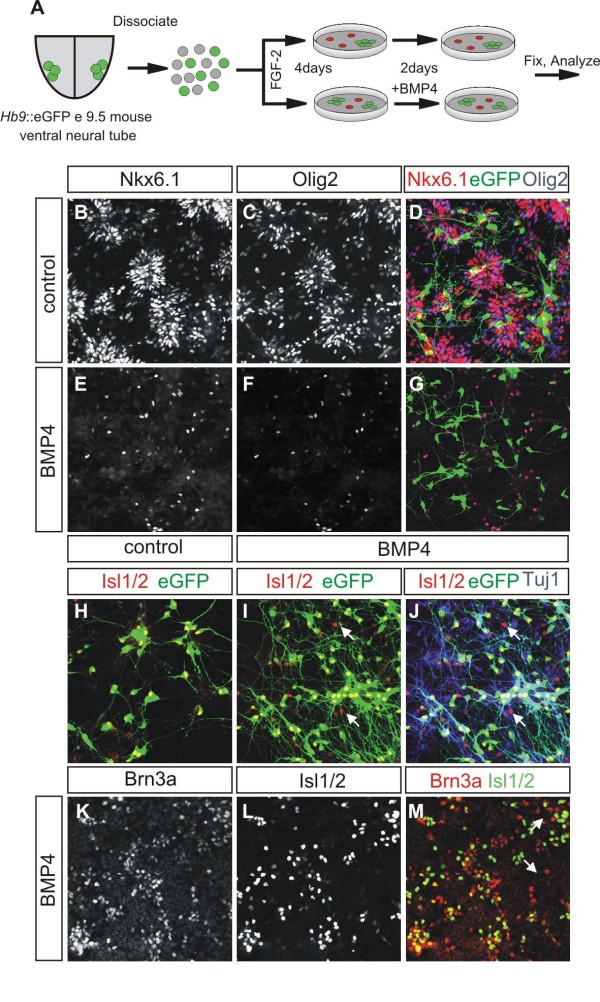
**Clones derived from a pMN-restricted progenitor are not committed to the motor neuron fate**. **(A) **Diagram of the experimental strategy used to test the commitment of pMN-restricted clones. **(B-G) **Staining of pMN-restricted clones for Nkx6.1 (B, E), Olig2 (C, F) and merge panels with Nkx6.1 (red), Olig2 (blue), and eGFP (green) (D, G). Nkx6.1 and Olig2 label motor neuron progenitors in control (B-D) and BMP4-treated (E-G) clones. Both Nkx6.1 and Olig2 are downregulated in BMP4-treated motor neurons clones. eGFP labels MNs. **(H-J) **Staining of MNs for Isl1/2 and eGFP in control (H) and BMP4-treated clones (I, J). Several Isl1/2^+ ^Tuj1^+ ^neurons are not labeled with eGFP in BMP4-treated cultures (I, J; white arrows). **(K-M) **Immunhistochemistry for Brn3a (K), Isl1/2 (L) and merge panel (M) of BMP4-treated MN clones. Some Isl1/2^+ ^neurons also express Brn3a (M; yellow cells, white arrow).

We next tested if lineage-restricted ventral progenitor subtypes that can faithfully transmit their transcriptional identity to their progeny are also committed to generate only ventral neuronal subtypes when challenged with a dorsal fate-inducing signal. For this we used the secreted molecule Bone Morphogenetic Protein (BMP4), a member of the Transforming Growth Factor-β superfamily, which is expressed in the roof plate and directs the specification of dorsal progenitor (pd1–pd3) and neuronal cell types (D1–D3) [38,39]. We dissociated ventral spinal cords from e9.5 *Hb9*::*eGFP *mice and cultured progenitors at clonal density for 4 days in the presence of FGF2, to allow formation of clones. After this period, half of the clones were exposed to BMP4 (20 ng/ml) for two additional days then analyzed (Figure 7A). We focused our analysis on clones that had been derived from pMN-restricted progenitors, since we could follow their progeny in live cultures by virtue of eGFP expression in MNs [30].

pMN-restricted clones were identified by expression of Nkx6.1 and Olig2 in progenitors and eGFP in MNs (Figure 7B–D). Upon exposure to BMP4, the expression of Nkx6.1 and Olig2 was downregulated in these clones, which could still be identified by the presence of neuronal eGFP (Figure 7E–G). In addition, many Isl1/2^+ ^eGFP^- ^neurons were detected in the BMP4-treated clones (Figure 7I,J). By contrast, in clones grown only in the presence of FGF2, all Isl1/2^+ ^neurons expressed eGFP (Figure 7H). We found that several Isl1/2^+ ^GFP^- ^neurons in BMP4-treated clones also expressed the transcription factor Brn3a (Figure 7K–M), which is normally found in dorsal Isl1/2^+ ^D3 interneurons [40]. However, we did not detect any Lhx2/9^+ ^D1 interneurons, which represent the dorsalmost neuronal subtype in the spinal cord [38], in clones cultured with BMP4 (data not shown). This could either reflect the fact that higher concentrations of BMPs are required for induction of Lhx2/9^+ ^neurons [38], or that MN progenitors cannot assume all dorsal fates due to an intrinsic restriction in their potential. However, when we treated Nkx6.1-patchy clones with the same amount of BMP4, many Lhx2/9^+ ^D1 neurons were generated (Figure S4D-I in Additional file 4). Therefore, the inability of pMN-restricted clones to produce D1 neurons upon exposure to BMP4 may reflect a restriction in their developmental potential. Taken together, these data indicate that pMN-restricted progenitors are not committed to their ventral fate and can acquire some dorsal fates upon exposure to BMPs.

In summary, our studies have revealed an unanticipated degree of heterogeneity among neuronal progenitors within a transcriptionally defined domain, with respect to their ability to maintain a dorsoventral and rostrocaudal identity and to generate appropriate neuronal subtypes when expanded *in vitro*. Subtype-restricted progenitors are present in all three progenitor domains that constitute the Nkx6.1^+ ^region of the spinal cord. These progenitors can maintain their identity for extended periods in culture in the absence of inductive signals. However, they are not absolutely committed and can acquire alternative fates upon exposure to dorsal signals.

## Discussion

In this study, we have asked whether pre-patterned spinal progenitors, in particular pMN progenitors, have the ability to maintain their positional identity after *in vitro *expansion. To achieve this we generated an *Nkx6.1::IRES::eGFP *mouse strain that expresses *eGFP *in the three populations of ventral spinal cord progenitors derived from the Nkx6.1^+ ^region. We have purified these progenitors by FACS after dorsoventral patterning is established and distinct progenitor domains have emerged, and determined the molecular identity of their clonal progeny after *in vitro *expansion. We demonstrate that subtype-restricted progenitors from all three progenitor domains are present in the spinal cord, although at low frequency. However, these progenitors are not committed to their ventral fates and can adopt alternative fates when exposed to dorsal fate-inducing signals. The low frequency of progenitor-restricted subtypes provides the first evidence for an unanticipated heterogeneity in the developmental potential of progenitors within a transcriptionally defined domain, despite seemingly uniform expression of the characteristic transcription factors at the time of isolation. We discuss these findings with respect to two major issues: lineage restrictions in the spinal cord and the timing of intrinsic cell identity programs within neural progenitors; and pattern formation and implications for the developmental potential of isolated CNS stem cells or progenitors.

### The identification of lineage-restricted progenitor subtypes and their emergence in the spinal cord

The onset of HD and bHLH protein expression by neural progenitors is a critical step in ventral patterning [1]. The majority of these proteins function as transcriptional repressors [41], and their mutual cross-repressive interactions are crucial for the establishment of progenitor domains within the neural tube [20]. Despite a detailed understanding of this process, it is unclear whether patterned spinal progenitors, in particular pMN progenitors, can maintain their regional identity after expansion in culture. This finding has significant implications for developing methods geared towards the production of large numbers of progenitors and MNs required for treating neurodegenerative diseases in the clinic. Moreover, it is not known if pMN progenitors are restricted with respect to the fates that they can acquire. Recent studies have shown that freshly sorted Olig2^+ ^progenitors are not restricted when transplanted into the chick neural tube [42]. However, only a small fraction of FACS-purified Olig2^+ ^progenitors were able to graft in that case, and it is possible, therefore, that some Olig2^+ ^cells may in fact be subtype-restricted progenitors.

Our molecular analysis of clones derived from single sorted Nkx6.1::eGFP cells has revealed the presence of subtype-restricted ventral progenitors from all three populations normally found within the Nkx6.1^+ ^region, namely the p2, pMN and p3 progenitors. We provide the first evidence that only a subset of ventral progenitors that have received positional information can subsequently divide and transmit positional identities to their clonal progeny after *in vitro *expansion. Importantly, these progenitors are not committed to their ventral fate. They can acquire some, although not all, dorsal fates upon exposure to a dorsal fate-inducing signal, thus revealing a restriction in fate acquisition that is consistent with other studies [42]. Interestingly, the pMN-restricted progenitors acquire a uniform rostrocaudal thoracic identity in culture, as revealed by expression of HoxC9, despite their diverse origins from both brachial and thoracic segments. These results are not surprising in light of the established role for FGF signaling in both the expression of Hox genes and the assignment of rostrocaudal identities of MNs within the spinal cord [25-27]. Finally, pMN-restricted progenitors have a limited proliferating and neurogenic capacity in culture, and, therefore, their expansion using current methods is not adequate for generating a sufficient number of cells for replacement therapies. CNS endothelial cells are known to secrete factors that stimulate self-renewal and prolong neurogenesis of isolated cortical progenitors [43]. Analogous factors may enhance proliferation and promote production *in vitro *of MNs within pMN-restricted progenitors. Our study is reminiscent of *in vitro *time-lapse lineage analyses of single mammalian cortical [12,13] and retinal [14] progenitors and *Drosophila *neuroblasts [44], which have revealed the importance of cell-intrinsic mechanisms for generating the correct temporal order of neurons. These studies, combined with our findings, emphasize the general role that intrinsic mechanisms play in neuronal subtype specification in the developing CNS for both invertebrates and vertebrates.

When are subtype-restricted progenitors generated in the spinal cord? Distinct neural progenitor domains emerge at approximately e9.5 in the mouse spinal cord [20,21,23], when ventral progenitors undergo multiple rounds of cell division [45] and are exposed to the Shh gradient [46,47]. All Nkx6.1^+ ^neural progenitors express apparently homogeneous levels at e9.5–e10 of the transcription factors characteristic of the three progenitor domains [20,23]. If these transcription factors are sufficient to confer lineage restrictions, then all progenitors should maintain their identities after *in vitro *expansion. However, we observed a strikingly heterogeneous behavior of Nkx6.1^+ ^progenitors after expansion, independent of when they were isolated. This heterogeneity could result from an asynchrony in cell division cycles among progenitors [45], such that some are exposed for a longer time to Shh signaling than others and, as a result, have higher concentrations of intrinsic factors that define their regional identities. We postulate that, when a critical threshold of these factors is achieved within progenitors, cell-intrinsic mechanisms are activated that stably maintain their expression in progenitors, as reflected in the maintenance of progenitor identity *in vitro*. This model suggests that there are heterogenous levels of transcription factors within cycling progenitors from a given domain during development, although to date these have been difficult to substantiate using fluorescent immunohistochemistry [20,23].

One prediction from our model is that the Nkx6.1^+ ^progenitors exposed to Shh signaling for the longest time will give rise to Nkx6.1-positive clones when expanded *in vitro*, since the levels of intrinsic factors are above a threshold necessary for maintaining their identities. Nkx6.1^+ ^progenitors that have been exposed for a shorter time, on the other hand, contain levels of intrinsic factors that are below the necessary threshold and will lose their identities when deprived of inductive signals. The latter category most likely gives rise to the Nkx6.1-negative and Nkx6.1-patchy clones observed in culture, and our clonal analysis at three developmental time points supports this model. The fraction of Nkx6.1^+ ^progenitors that generate Nkx6.1-positive clones increases dramatically over the 12-hour period from e9.5 to e10.0, whereas Nkx6.1^+ ^progenitors that cannot maintain their identities are present at decreasing frequencies over this period. We have attempted to culture single Nkx6.1^+ ^progenitors in the presence of Shh, to determine if this would promote the Nkx6.1-positive subtype in the resulting clones. However, Shh induced differentiation of neural progenitors, precluding further analysis (DA and IS, unpublished data). Finally, although we cannot exclude the possibility that autocrine signals secreted by a subset of Nkx6.1^+ ^progenitors reinforce their progenitor identities, Shh is an unlikely candidate for such a signal since it is not secreted by neural progenitors in culture (DA and IS, unpublished observations).

How can transcription factors regulate the maintenance of neuronal progenitor identity in culture? The majority of class II HD proteins functions in neural progenitors as transcriptional repressors that recruit a general co-repressor to regulate in turn the expression of progenitor HD proteins and subtype determinants [20,23,41]. In addition, various Nkx6 proteins have repressive roles with respect to other progenitor HD proteins or neural subtype determinants during the acquisition of ventral cell fates [21]. Therefore, high levels of Nkx6.1 and Olig2 in some progenitors may enable them to suppress genes that would otherwise block their transcription and the acquisition of ventral fates. One potential target would be repressors that are induced in response to activated FGF signaling in culture and that inhibit the expression of HD and bHLH proteins [48,49]. In addition, chromatin modifications that regulate active or repressed states of gene expression [50] can be transiently lost during cell division [51,52] and may change during progenitor maturation [53]. This could also explain the sensitivity of cells during cell division to signals that affect CNS regional patterning *in vivo *and *in vitro *[54-57].

### Neural patterning and the acquisition of identity by isolated CNS stem or progenitor cells

Studies of embryonic neural pattern formation in the CNS have revealed that the process of neuronal subtype determination begins with regional specification of progenitors within the ventricular zone [1,2,58,59]. Progenitors from different regions of the CNS express defined combinations of transcription factors in response to morphogens such as Shh, BMPs, FGFs and Wnts. Genetic studies have revealed essential roles for several transcription factors in cell fate decisions, in both the cortex and spinal cord. With the identification of neural stem cells in the adult brain and successful prospective isolation *in vitro *of embryonic and adult neural progenitors [60-66], one of the major challenges has been to understand if isolated stem cells and progenitors maintain a cellular memory of their origin, as this may have implications for understanding their developmental potential and therapeutic uses [4,67].

Conflicting results have been reported on this issue, with some studies claiming that the majority of neural stem cells or progenitors isolated as early as e14.5 or as late as the adult, from different regions of the CNS, can maintain the expression of region-specific transcription factors when grown *in vitro *as neurospheres [68-73]. On the other hand, conservation of gene expression has not been detected in some other studies [74-77], and progenitors grown in the presence of mitogens such as FGF2 or Epidermal Growth Factor (EGF) can undergo reprogramming and expand their developmental potential [76,78,79]. One potential mechanism for this plasticity involves chromatin remodeling through repression of histone deacetylases, which expands the developmental potential of isolated progenitors or stem cells [57,80]. Alternative models have attributed the expansion of developmental potential to the ability of FGF2 to deregulate expression of regionally restricted transcription factors within neural progenitors [77].

Our findings have important implications for linking regional identity to the amplification of progenitors *in vitro*. First, we observed that the expression of class I transcription factors (for example, Irx3, Pax6) was lost in all clones derived from Nkx6.1^+ ^progenitors in culture, and there was variable expression of class II proteins (Nkx6.1, Nkx2.2 and Olig2) in these clones, although they were derived from single cells that initially expressed these transcription factors. In addition, most forelimb-derived pMN-restricted clones change their Hox expression profile after *in vitro *expansion and acquire rostrocaudal identities characteristics of more posterior (thoracic) MNs. Together, these findings suggest that FGF2 can actively deregulate expression of several transcription factors expressed by neural progenitors, with class I HD proteins being most severely affected, as is also observed in other studies [74-77]. This suggests that dorsal progenitors are more likely than ventral progenitors to lose their regional identity when grown in the presence of FGF2. Second, heterogeneity in Nkx6.1 expression within clones derived from Nkx6.1^+ ^cells may explain the discrepancies among several reported attempts to culture specific CNS progenitors. Most clones derived from an Nkx6.1^+ ^progenitor lose their ventral identity, even if they are isolated after dorsoventral patterning is established. Furthermore, these clones do not show any restrictions in the acquisition of neuronal fates from other regions of the dorsoventral neuraxis. Finally, the small percentage of lineage-restricted progenitors that we have identified are not committed to their identities, and can change fates upon exposure to dorsally derived signals. These findings may underlie the reprogramming and expansion of developmental potential for isolated CNS progenitors exposed to mitogens such as FGF and EGF [76,78,79].

## Conclusion

We have generated a mouse strain that expressed eGFP in ventral progenitors of the spinal cord. We have purified these progenitors by FACS after dorsoventral patterning is established and performed single cell clonal analysis *in vitro*, in order to test if CNS progenitors that have received positional information can maintain their cellular identity after *in vitro *expansion. We demonstrate that subtype-restricted progenitors from three spinal domains (p2, pMN and p3) are present in the spinal cord at a low frequency, and that they can transmit their subtype identities to their progeny. However, these cells are not committed to their fates and can adopt alternative fates when exposed to dorsally derived extracellular signals. The low frequency of progenitor-restricted subtypes provides the first evidence for an unanticipated heterogeneity in the behavior of progenitors within a domain, despite the apparently uniform expression of characteristic transcription factors at the time of isolation, and suggests that prolonged exposure of progenitors to Shh signaling is critical for the establishment of cell fates. Furthermore, most forelimb-derived pMN-restricted clones change their Hox expression profile after *in vitro *expansion and acquire new rostrocaudal identities that are characteristic of more posterior (thoracic) MNs. Together, these findings suggest that neural progenitors have a high degree of plasticity with respect to changing their dorsoventral and rostrocaudal identites after *in vitro *expansion.

## Materials and methods

### Generation of *Nkx6.1::IRES::eGFP *mice

We inserted an *IRES::eGFP::ACN *cassette [81] immediately after the *Nkx6.1 *stop codon to ensure gene-dependent expression of *eGFP*. We constructed an *Asc*I site in the *Nkx6.1 *3'-genomic fragment by inserting a 43 bp oligomer adapter (5'CGC GTC GGA GGC CGA GGG CTC GTC CTG AGG CGC GCC CCG CGC G3') into the *Mlu*I site located 46 bp upstream of the stop codon, in order to clone the selection cassette (Figure 1A). The targeting vector was electroporated in embryonic stem cells and three recombinant clones were identified by the presence of two fragments: a 15.5 kb recombined fragment and a 12.5 kb wild-type fragment (Figure 1B). Two of these recombinant clones were injected into blastocysts to generate chimeric mice. The ACN cassette was self-excised itself during male germ line transmission of the targeted allele, due to induced expression of Cre recombinase from the tACE promoter [81]. The *Nkx6.1::IRES::eGFP *knock-in homozygous animals were viable and were genotyped by PCR. The knock-in allele was screened for the presence of eGFP with the primers GFPF1 (5'CCC TGA AGT TCA TCT GCA CCA C3') and GFPR1 (5'TTC TCG TTG GGG TCT TTG CTC 3'), using an amplification protocol (94°C for 1 minute, 60°C for 30 s and 72°C for 1 minute; 30 cycles) to amplify a 500 bp product. The wild-type allele was screened with the primers Nkx6.1F1 (5' CCG ATG ACG AGA AGA TCA C3'), located 100 bp upstream of the stop codon, and Nkx6.1R1 (5'TCC TTT TCT CCT CAT CAG CG3'), located 180 bp downstream of the stop codon, for amplification of a 300 bp product. The following amplification protocol was used: 94°C for 1 minute, 57°C for 90 s and 72°C for 45 s, 30 cycles. The *HB9::eGFP *transgenic animals [30] were obtained from Ivo Lieberam. Animals were housed at the Columbia University Animal Facility and handled according to institutional guidelines (Animal Protocol # 1156).

### Dissociation and culture of ventral spinal cord progenitors

*Nkx6.1::IRES::eGFP*^+/- ^embryos were removed at e9.0, e9.5 or e10.0 and eGFP expression in the ventral spinal cord was confirmed by visualization under a fluorescence dissecting microscope. The neural tube with surrounding somites from the brachial and thoracic segments was dissected with a microsurgical knife (Surgical Specialties, Reading, PA, USA) and incubated in papain solution at 37°C for 30–35 minutes. The tissue was triturated with a P200 pipette at regular intervals (8–10 minutes) to mechanically break down the clumps and allow the enzyme to penetrate. The papain activity was inhibited by addition of culture medium containing 10% albumin-ovomucoid inhibitor. Cells were spun down at 300 g for 5 minutes and triturated 20 times to create a single-cell suspension. Finally, the pellet was resuspended in DMEM/F12 medium with 2% horse serum plus propidium iodide (2 μg/ml) for FACS. FACS was performed with a Beckmann Coulter Epics^® ^Altra™ Hypersort system (Fullerton, CA, USA). Cells were selected based on their forward and side scatter properties as well as propidium iodide exclusion. The GFP^+ ^fraction of these viable single cells was collected in Eppendorf tubes with culture medium, washed and plated in 72-microwell titer plates (Fisher Scientific, Pittsburgh, PA, USA) at a density of 8–10 cells/microwell.

*Hb9*::*eGFP*^+/- ^embryos were removed at e9.5 and eGFP expression in spinal MNs was confirmed by visualization under a fluorescence dissecting microscope [30]. The eGFP^+ ^ventral spinal cords were dissected away from the dorsal spinal cord and somites, transferred to an Eppendorf tube and dissociated into a single cell suspension with papain at 37°C for 20–30 minutes as described above. The dissociated cells were cultured under the same conditions as sorted eGFP^+ ^progenitors from *Nkx6.1::IRES::eGFP *mice.

Microwell titer plates were coated with poly-L-Lysine solution (0.01%; Sigma, St. Louis, MO, USA) for 1 hour. These plates were washed with sterile water and air-dried for 1 hour in the hood. Afterwards, plates were coated with dilute matrigel solution (BD Biosciences, Bedford, MA, USA) 1:30 dilution in phosphate-buffered saline (PBS) overnight at 4°C. The following day, the matrigel was washed with PBS and half of the microwell volume was filled with culture medium before plating. Cells were cultured in DMEM/F12 with 15 mM HEPES containing glucose, N2 and B27 supplements, 0.75% bovine serum albumin, N-acetyl cysteine (NAC) and FGF2 at 20 ng/ml. Cells were fed with fresh medium every 48 hours and the formation of clones from single cells was monitored every day with an inverted microscope. Wells that contained two or more clones in close proximity were not included in the clonal analysis. At the end of the culture period, the plates were washed with cold PBS and clones were fixed with cold 4% paraformaldehyde in 0.1 M phosphate buffer (PB) solution for 10 minutes at room temperature, rinsed three times with PBS and processed for immunohistochemistry. Other mitogens that we tested, such as EGF (10 ng/ml), or Shh agonist (ShhAg1.3 = 100 nM) [37] did not induce proliferation of Nkx6.1^+ ^progenitors.

### Immunohistochemistry

Immunohistochemistry on sections of embryos and cultured neural progenitor clones was performed as described [34]. The following primary antisera were used: rabbit anti-GFP (Molecular Probes, Eugene, OR, USA; 1:1000), sheep anti-GFP (Biogenesis, Kingston, NH, USA; 1:1,000), mouse anti-Pax6 (1:10), mouse anti-Pax7 (1:5), rabbit anti-HNF3β (K2; 1:4000), rabbit anti-mouse Nkx6.1 (1:4000), guinea pig anti-mouse Nkx6.2 (1:5000), rabbit anti-Brn3a (1:400), rabbit anti-Nkx2.2 (1:4000), mouse anti-Nkx2.2 (75-5A5; 1:50), rabbit anti-mouse Olig2 (1:16,000), guinea pig anti-mouse Olig2 (1:8000), rabbit anti-Hb9 (1:10,000), guinea pig anti-Hb9 (1:8000), rabbit anti-Lhx1/2 (1:4000), rabbit anti-Lhx2/9 (LH2A/B; 1:8000), rabbit anti-Lhx3 (1:4000), guinea pig anti-Lhx3 (1:4000), rabbit anti-Chx10 (1:4000), guinea pig anti-Isl1/2 (1:16,000), rat anti-Math3 (1:2000), rabbit anti-mouse Dbx1 (1:16,000), rabbit anti-mouse Dbx2 (1:8000), guinea pig anti-En1 (1:8000), guinea pig anti-Evx1/2 (1:8000), guinea pig anti-HoxC6 (1:4000), rabbit anti-HoxC9 (1:4000), rabbit anti-HoxD10 (1:4000), β3-Tubulin (TUJ1; 1:1000; Covance, Denver, PA, USA).

## Abbreviations

bHLH: basic helix-loop-helix; BMP: bone morphogenetic protein; CNS: central nervous system; DIV: days *in vitro*; e: embryonic day; EGF: epidermal growth factor; eGFP: enhanced green fluorescent protein; FACS: fluorescence-activated cell sorting; FGF: fibroblast growth factor; HD: homeodomain; IRES: internal ribosome entry site; MN: motor neuron; PBS: phosphate-buffered saline; s.e.m: standard error of the mean; Shh: Sonic Hedgehog.

## Competing interests

The authors declare that they have no competing interests.

## Authors' contributions

DA generated the *Nkx6.1::IRES::eGFP *mice, carried out the dissociation and culture of neural progenitors, analyzed the molecular identity of clones by immunofluorescence, counted the number of clones, created most of the figures and wrote the manuscript. IS performed FACS analysis of dissociated progenitors, analyzed their purity after FACS isolation and helped to create Figures 2, 4, 6 and S2.

## Supplementary Material

Additional file 1 **Nkx6.1-derived progenitors that lose ventral identity do not acquire dorsal identities *in vitro***. **(A-H) **Expression of Nkx6.1 (A, C, E, G), Olig2 (B), Nkx6.2 (D), Dbx2 (F) and Pax7 (H) in Nkx6.1-patchy clones that gradually lose their ventral identities. Note the patchy expression of Nkx6.1 (A) and Olig2 (B) in this clone. No expression of Pax7, Dbx2 or Nkx6.2 was detected in any clone (n = 60 clones). **(I-K) **Immunohistochemistry for Nkx6.1, Hb9 and Tuj1 of Nkx6.1-patchy clones. A subset of progenitors is labeled with Nkx6.1 (I); a subset of neurons is Hb9^+ ^MNs (J, K). **(L-N) **Staining for Lim1/2 (dorsal neuronal marker) (L), Hb9 (M) and merge panel (N) in presumed Nkx6.1-patchy clones. Note that Hb9-negative neurons do not express Lim1/2 (white arrow). **(O-Q) **Clones derived from pMN progenitors in *Hb9*::eGFP transgenic mice. This clone generated motor neurons first (O; green), followed by other types of neurons (black arrows). Motor neurons expressing eGFP (green) and Isl1/2 (red) (P) are only a subset of total neurons in this clone (Q). Click here for file

Additional file 2 **Expression of eGFP and sorting of Nkx6.1 progenitors from e9.0 and e10.0 neural tube of *Nkx6.1::IRES::eGFP*^+/- ^mice**. **(A, D) **Contour plots for cells dissociated from e9.0 (A) and e10.0 (D) trunks of *Nkx6.1::IRES::eGFP*^+/- ^mice. The logarithmic scale of eGFP fluorescence is represented on the x-axis and cell size on the y-axis (forward scatter); 5.9% of cells are eGFP^+ ^at e9.0 and 18% of cells are eGFP^+ ^at e10.0. **(B, C, E, F) **Immunohistochemistry for eGFP (B, E) and Nkx6.1 (C, F) proteins in the e9.0 and e10.0 neural tubes of *Nkx6.1::IRES::eGFP*^+/- ^mice. eGFP is expressed in Nkx6.1^+ ^progenitors (e9.0 and e10.0) and neurons (e10.0). Click here for file

Additional file 3 **pMN-restricted progenitors generate oligodendrocytes after a prolonged culture period**. **(A-D) **Expression of O4 (A), Nkx6.1 (B), Olig2 (C) and merge panel (D) in clones derived from pMN-restricted progenitors after 12 days *in vitro *(DIV). Note that many O4^+ ^oligodendrocytes also express Olig2 in these clones. Click here for file

Additional file 4 **Nkx6.1-patchy clones generate dorsal neuronal subtypes in the presence of dorsal fate-inducing signals.(A-C) **Labeling of a Nkx6.1-patchy clone with Hb9 (A), Isl1/2 (B) and merge panel (C) with Hb9 (blue), Isl1/2 (red) and Tuj1 (green). Very few neurons are MNs. **(D-I) **Patchy clones were exposed to BMP4 for 48 hours prior to analysis. BMP4 generates many Isl1/2^+ ^neurons (E, F; red cells) that do not express Hb9 (D). In addition, BMP4 induces some Lhx2/9^+ ^D1 neurons (H, I). No Math1^+ ^progenitors were detected. **(J-L) **Retinoic acid (0.5 μM) induces generation of V1 interneurons expressing En-1 (J) and Lim1/2 (K). Many En-1 neurons are also labeled with Lim1/2 (L; merge panel). Click here for file
