# Supplementary figures and images for: Heterogeneity in the developmental potential of motor neuron progenitors revealed by clonal analysis of single cells in vitro
Source: Neural Dev. 2009 Jan 5;4:2. doi: 10.1186/1749-8104-4-2 (PMC2657897; doi:10.1186/1749-8104-4-2)

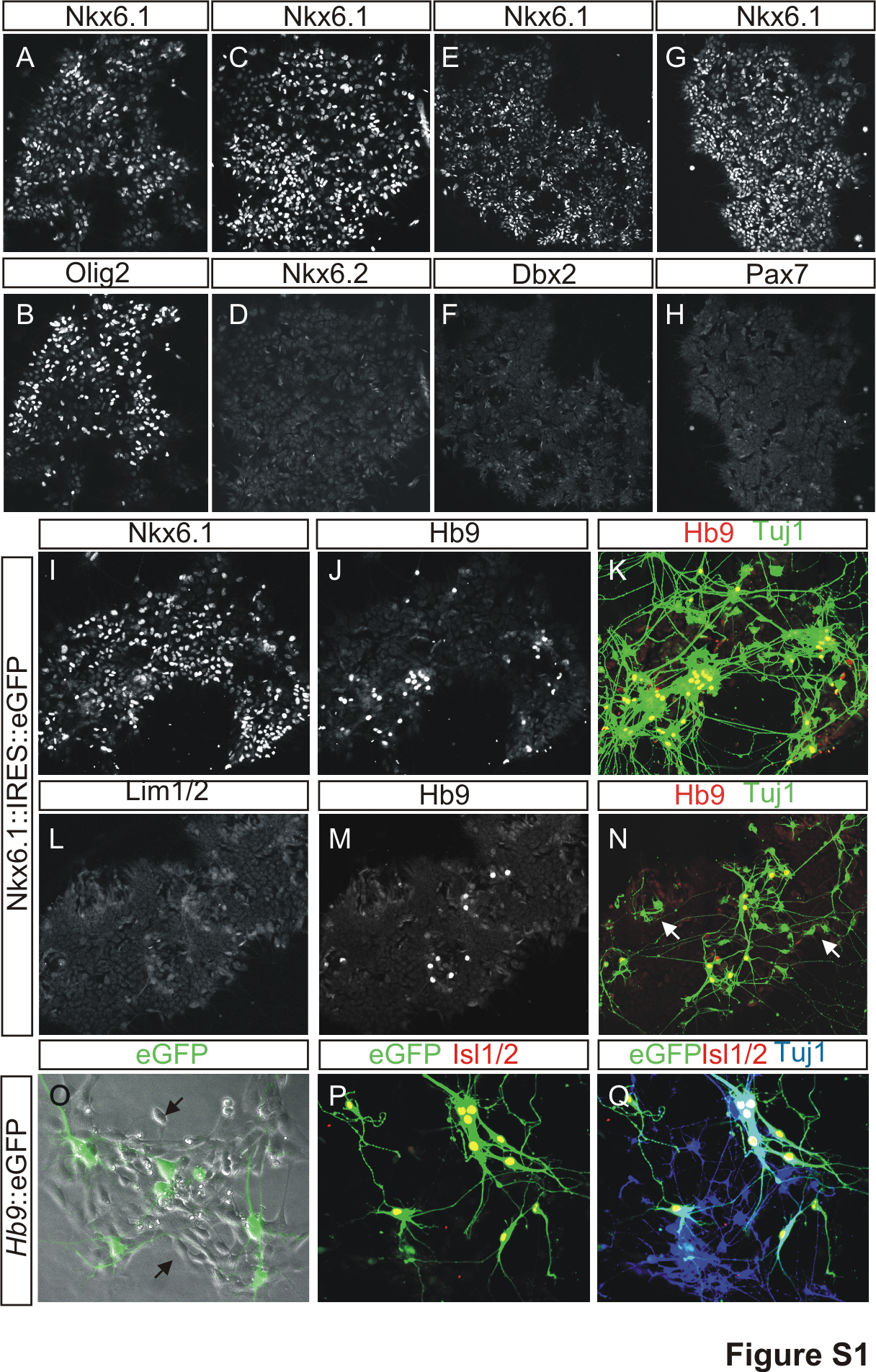

Supplement: Additional file 1 — Nkx6.1-derived progenitors that lose ventral identity do not acquire dorsal identities in vitro. (A-H) Expression of Nkx6.1 (A, C, E, G), Olig2 (B), Nkx6.2 (D), Dbx2 (F) and Pax7 (H) in Nkx6.1-patchy clones that gradually lose their ventral identities. Note the patchy expression of Nkx6.1 (A) and Olig2 (B) in this clone. No expression of Pax7, Dbx2 or Nkx6.2 was detected in any clone (n = 60 clones). (I-K) Immunohistochemistry for Nkx6.1, Hb9 and Tuj1 of Nkx6.1-patchy clones. A subset of progenitors is labeled with Nkx6.1 (I); a subset of neurons is Hb9+ MNs (J, K). (L-N) Staining for Lim1/2 (dorsal neuronal marker) (L), Hb9 (M) and merge panel (N) in presumed Nkx6.1-patchy clones. Note that Hb9-negative neurons do not express Lim1/2 (white arrow). (O-Q) Clones derived from pMN progenitors in Hb9::eGFP transgenic mice. This clone generated motor neurons first (O; green), followed by other types of neurons (black arrows). Motor neurons expressing eGFP (green) and Isl1/2 (red) (P) are only a subset of total neurons in this clone (Q). [file 1749-8104-4-2-S1.tiff]

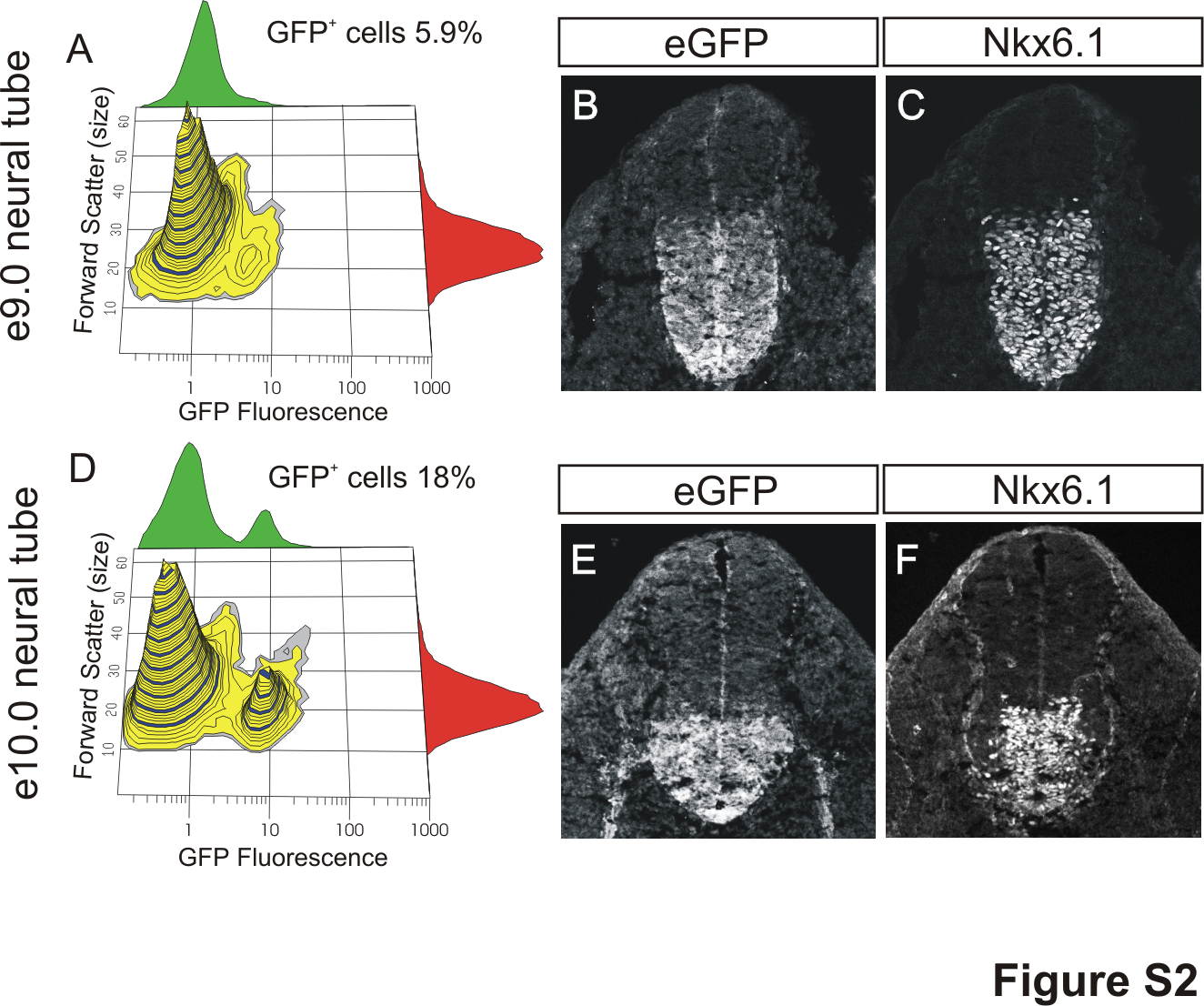

Supplement: Additional file 2 — Expression of eGFP and sorting of Nkx6.1 progenitors from e9.0 and e10.0 neural tube of Nkx6.1::IRES::eGFP+/- mice. (A, D) Contour plots for cells dissociated from e9.0 (A) and e10.0 (D) trunks of Nkx6.1::IRES::eGFP+/- mice. The logarithmic scale of eGFP fluorescence is represented on the x-axis and cell size on the y-axis (forward scatter); 5.9% of cells are eGFP+ at e9.0 and 18% of cells are eGFP+ at e10.0. (B, C, E, F) Immunohistochemistry for eGFP (B, E) and Nkx6.1 (C, F) proteins in the e9.0 and e10.0 neural tubes of Nkx6.1::IRES::eGFP+/- mice. eGFP is expressed in Nkx6.1+ progenitors (e9.0 and e10.0) and neurons (e10.0). [file 1749-8104-4-2-S2.tiff]

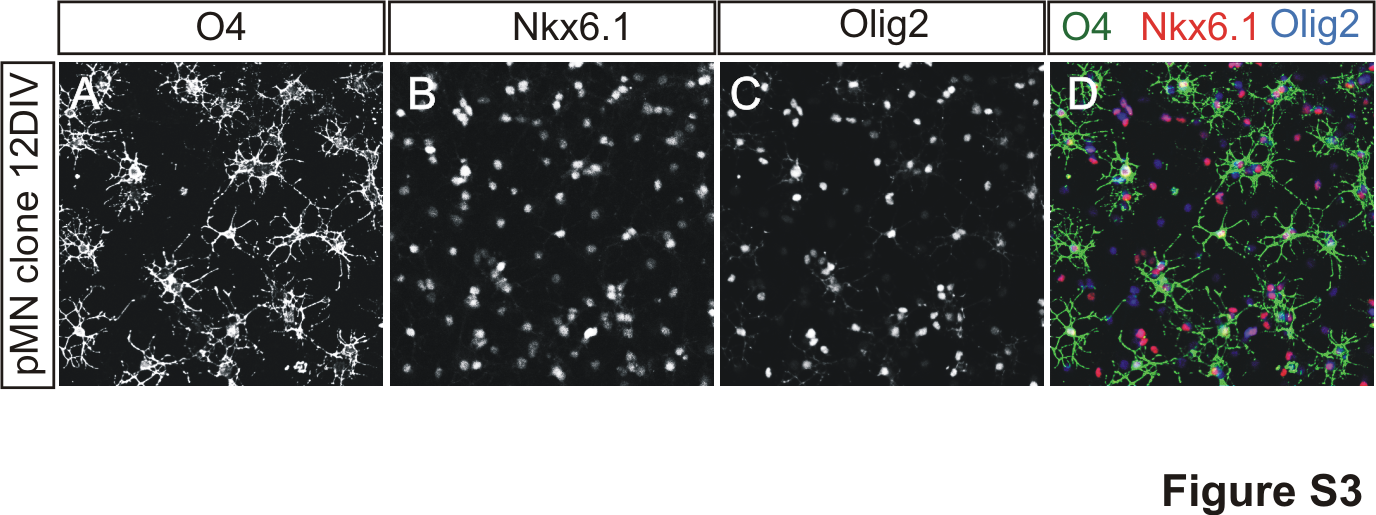

Supplement: Additional file 3 — pMN-restricted progenitors generate oligodendrocytes after a prolonged culture period. (A-D) Expression of O4 (A), Nkx6.1 (B), Olig2 (C) and merge panel (D) in clones derived from pMN-restricted progenitors after 12 days in vitro (DIV). Note that many O4+ oligodendrocytes also express Olig2 in these clones. [file 1749-8104-4-2-S3.tiff]

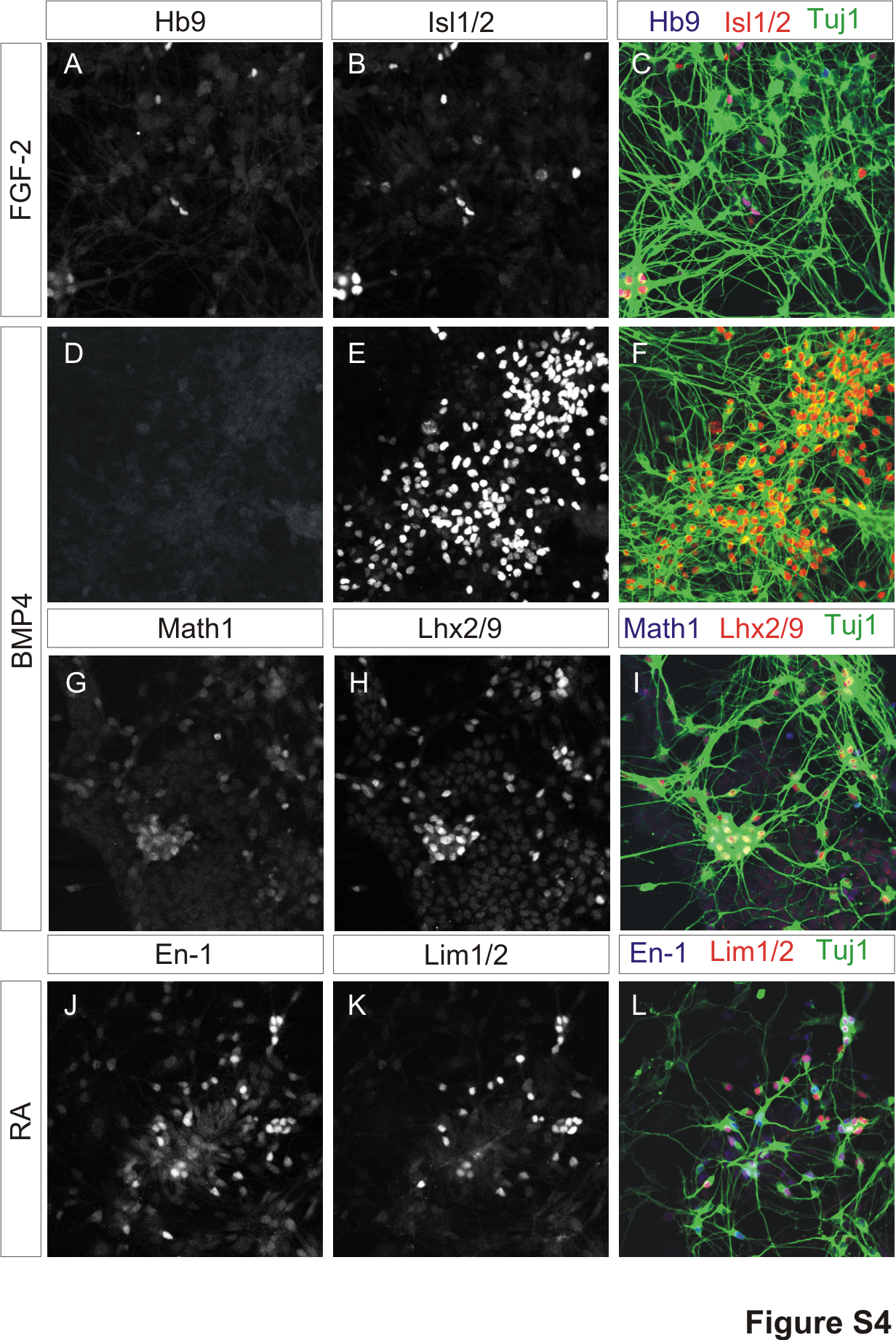

Supplement: Additional file 4 — Nkx6.1-patchy clones generate dorsal neuronal subtypes in the presence of dorsal fate-inducing signals.(A-C) Labeling of a Nkx6.1-patchy clone with Hb9 (A), Isl1/2 (B) and merge panel (C) with Hb9 (blue), Isl1/2 (red) and Tuj1 (green). Very few neurons are MNs. (D-I) Patchy clones were exposed to BMP4 for 48 hours prior to analysis. BMP4 generates many Isl1/2+ neurons (E, F; red cells) that do not express Hb9 (D). In addition, BMP4 induces some Lhx2/9+ D1 neurons (H, I). No Math1+ progenitors were detected. (J-L) Retinoic acid (0.5 μM) induces generation of V1 interneurons expressing En-1 (J) and Lim1/2 (K). Many En-1 neurons are also labeled with Lim1/2 (L; merge panel). [file 1749-8104-4-2-S4.tiff]
